# How Sensitive is Sensitivity Analysis?: Evaluation of Pharmacoeconomic Submissions in Korea

**DOI:** 10.3389/fphar.2022.884769

**Published:** 2022-05-16

**Authors:** SeungJin Bae, Joohee Lee, Eun-Young Bae

**Affiliations:** ^1^ Ewha Womans University, College of Pharmacy, Seoul, Korea; ^2^ Gyeongsang National University, College of Pharmacy, Jinju, Korea

**Keywords:** economic evaluation, uncertainty, structural uncertainty, parametric uncertainty, sensitivity analysis, incremental cost effectiveness ratio

## Abstract

**Purpose:** We aimed to describe the types of uncertainties examined in the economic evaluations submitted for reimbursement in Korea and their impact on the incremental cost-effectiveness ratio (ICER).

**Method:** Fifty dossiers were submitted by pharmaceutical companies to the economic subcommittee of the Pharmaceutical Benefit Coverage Advisory Committee (PBCAC) from January 2014 to December 2018. The types of uncertainties were categorized as structural and parametric, and the frequencies of the sensitivity analysis per variables were analyzed. The impact of uncertainties was measured by the percent variance of the ICER relative to that of the base case analysis.

**Results:** Of the 50 submissions, varying discount rate (44 submissions), followed by time horizon (38 submissions) and model assumptions (29 submissions), were most frequently used to examine structural uncertainty, while utility (42 submissions), resource use (41 submissions), and relative effectiveness (26 submissions) were used to examine parametric uncertainty. A total of 1,236 scenarios (a scenario corresponds to a case where a single variable is varied by a single range) were presented in the one-way sensitivity analyses, where parametric and structural sensitivity analyses comprised 679 and 557 scenarios, respectively. Varying drug prices had the highest impact on ICER (median variance 19.9%), followed by discount rate (12.2%), model assumptions (11.9%), extrapolation (11.8%), and time horizon (10.0%).

**Conclusions:** Variables related to long-term assumptions, such as model assumptions, time horizon, extrapolation, and discounting rate, were related to a high level of uncertainty. Caution should be exercised when using immature data.

## Introduction

Model-based analysis synthesizes clinical, economical, and epidemiological evidence from various sources and extrapolates the expected value over the long term (Briggs et al., 2012). Since the evidence directly related to the research question is frequently missing, or multiple sources are available with conflicting results, model-based analysis almost always suffers from various forms of uncertainties (Bilcke et al., 2011).

Uncertainties can be classified as stochastic uncertainty (first-order), parametric uncertainty (second-order), structural uncertainty, and heterogeneity (Briggs et al., 2012). Stochastic uncertainty implies random variability and is intrinsically unavoidable, whereas parametric uncertainties imply the uncertainties in parameter estimation and can be examined via deterministic sensitivity analysis (DSA), where parameter values are varied based on defensible ranges of values to examine the robustness of the results, or via probabilistic sensitivity analysis (PSA), where parameter values are sampled from a predefined distribution and vary simultaneously (Doubilet et al., 1985). Structural uncertainty, which is inherent in the assumptions of the decision model, is a difficult type of uncertainty to define and can be examined by scenario analysis. Heterogeneity deals with the variability of patients of interest and is usually explored through a subgroup analysis. Interestingly, recommendations on how to tackle structural uncertainties or heterogeneity are largely vague in many international pharmacoeconomic guidelines, including Korea, while several provide specific details on parametric uncertainties (Ghabri et al., 2018).

Structural uncertainties are associated with a wide variation in the incremental cost-effectiveness ratio (ICER) (Le, 2016), and given that clinical trials usually last shorter than expected in the economic evaluation, extrapolating beyond the time horizon is frequently required, which introduces additional uncertainties (Kearns et al., 2020). When policymakers set priorities among competing demands based on model-based analysis, uncertainties related to the point estimates, how those uncertainties were examined or reported, and variance of the ICERs are deeply considered (Jackson et al., 2011; Bae et al., 2013), few studies have described how sensitivity analyses in the dossiers submitted for the reimbursement decision are handled, including parametric and structural uncertainties, much less the variance of ICER relative to the base case.

Many HTA organizations review DSA as well as PSA due to the advantage of being able to transparently check the effect of uncertainty of each variable on the results (Australian Government: Department of Health, 2016; The Canadian Agency for Drugs, 2017; The National Institute for Health and Care Excellence (NICE), 2013), Yet DSA, especially one-way SA, has a problem of underestimating the overall uncertainty because it examines only the effects of variations that one variable can have while other variables are fixed (Claxton, 2008). In addition, there is a limitation that the nonlinearity of the model is not reflected, and if there is a correlation between variables, it cannot be considered appropriately (Briggs et al., 2012; McCabe et al., 2020; Vreman et al., 2021). Moreover, despite many guidelines stipulated that that “clinically and statistically feasible ranges” are recommended, the ranges of the DSA are often chosen arbitrarily (Vreman et al., 2021).

This study examines how uncertainty is explored in economic evaluations submitted by pharmaceutical companies in Korea, which is a necessary part of the reimbursement decision-making process. Specifically, we examine how variables related to long-term effects are analyzed. Additionally, the impact of uncertain variables on ICER was explored through variation in the ICER.

## Methods

Economic evaluation dossiers submitted by the pharmaceutical industry to the economic subcommittee of the Pharmaceutical Benefit Coverage Advisory Committee from January 2014 to December 2018 were evaluated by two independent reviewers (SB and EB).

Uncertainties were categorized as DSA and PSA, and DSA was further categorized into structural and parametric uncertainties. Stochastic uncertainty, which is intrinsically unavoidable, and heterogeneity, which is known variability, are not included in the analysis. The number of sensitivity analyses per submission is used to identify the frequently tested parameters or structural assumptions. The parametric uncertainties considered in our study are drug prices, resource use (unit cost or resource utilization other than drug), utility weights, relative effectiveness of the intervention (including odds ratio, relative risk, or hazard ratio), baseline risk (natural history of the disease not related to specific treatment), and others (parameters relevant to specific treatment, such as incidence of the adverse event). The plausible range of values used in the sensitivity analysis is categorized as a 95% confidence interval (CI) of a specific parameter (statistically obtained from clinical studies), arbitrarily selected values (± 20%), or sourced from other studies.

The structure of a model varies by disease type, yet we refer to variables that are universally applicable and can be clearly defined, such as time horizon, discount rate [variation from the recommended 5% (Bae et al., 2013)], extrapolation method used (i.e., Weibull vs. lognormal), model assumptions [i.e., treatment duration, duration of the effectiveness, selection of comparator(s)], and patient characteristics (i.e., age, disease severity, weight, or race).

The frequency of the sensitivity analysis in this study is estimated on a submission or scenario basis. When presenting the proportion of submissions with sensitivity analysis for each category of variables, it is analyzed per submission, and the frequency of sensitivity analysis for each variable is analyzed for each scenario. To examine the ranges of the values used and their ICERs relative to the base case, we count scenarios; a single scenario for the sensitivity analysis corresponds to a case where a single parameter is varied by a single plausible range. A paired case (i.e., ± 20%) is also defined as a single scenario.

The variance of the ICER related to the sensitivity analyses is measured in percentage,

$$\frac{\left|{ICER}_{sensitivity\;analysis}{-ICER}_{base\;case}\right|}{{ICER}_{base\;case}}\text{×}100\text{,}$$

where paired (±95% CI) values are estimated as follows:

$$\frac{{\text{ICER}}_{\text{max}}{-\text{ICER}}_{\text{min}}}{{2\text{×ICER}}_{\text{base}\;\text{case}}}\text{×}100\text{.}$$

## Results

Of the 50 dossiers submitted to the economic subcommittee, 26 (52%) fall under antineoplastic and immunomodulating agents, and 24 (48%) are injection formulations and were submitted evenly across the observation period (Table 1). 46 submissions (92%) employed cost-utility analysis, and DSA was conducted in 49, all of which conducted one-way sensitivity analysis (Table 2), and only two of them conducted multivariate (2-way) sensitivity analysis (data not shown). Regarding structural uncertainties, the discount rate was the most frequently examined (44 submissions), followed by the time horizon (38 submissions), and model assumptions (29 submissions) (Table 2). For parametric uncertainty, utility was most frequently used (42 submissions), followed by resource use (41 submissions), and relative effectiveness (26 submissions) (Table 2). PSA was conducted in 18 submissions and cost was most frequently examined (17 submissions), followed by utility (16 submissions) and relative effectiveness (10 submissions).

**TABLE 1:** Basic characteristics of the 50 dossiers submitted to the Economic Sub-Committee for listing at the Korean National Health Insurance.

Variables	Submissions	
	*n*	%
WHO ATC Code^1^		
A	3	6
B	2	4
C	3	6
D	1	2
H	1	2
J	3	6
L	26	52
M	2	4
N	4	8
R	5	10
Formulation		
Gel	1	2
Tablet	13	26
Capsule	7	14
Injection	24	48
Spray	1	2
Pen	3	6
Inhaler	1	2
Submission Date		
2014	10	20
2015	8	16
2016	8	16
2017	12	24
2018	12	24
Types of Economic evaluation		
CEA^2^ only	2	4
CUA^3^ only	18	36
CEA & CUA	28	56
CMA^4^ only	2	4
Total	50	100

1 A, Alimentary tract and metabolism; B, Blood and blood forming organ; C, Cardiovascular system; D, Dermatologicals; H, Systemic hormonal preparations excl. sex hormones and insulins; J, Antiinfectives for systemic use; L, Antineoplastic and immunomodulating agents; M, Musculo-skeletal system; N, Nervous system; R, Respiratory system; S, Sensory organs

2 CEA, cost-effectiveness analysis.

3 CUA, cost-utility analysis.

4 CMA, cost-minimization analysis.

**TABLE 2:** Types of sensitivity analysis of the 50 dossiers examined.

Variables	Submissions	
	*n*	%
Sensitivity Analysis		
Deterministic sensitivity analysis	49	98
Probabilistic sensitivity analysis	18	36
No Sensitivity Analysis	1	2
Deterministic sensitivity analysis (*n* = 49)		
Structural Uncertainty		
Discount rate	44	90
Time horizon	38	71
Model assumptions	29	59
Extrapolation	19	39
Patient characteristics	19	39
Parameter Uncertaint ***y***		
Utility	42	86
Resource use	41	84
Relative effectiveness	26	53
Drug Price	16	33
Baseline risk	16	33
Other^1^	15	31
Total	49	100
Probabilistic sensitivity analysis (*n* = 18)		
Cost	17	94
Utility	16	89
Relative effectiveness	10	56
Baseline risk	8	44
Other^2^	9	58
Total	18	100

1 Parameters relevant with specific treatment, such as the incidence of the adverse event, or hospitalization rate.

2 Probability of discontinued treatment.

A total of 1,236 scenarios were presented in the one-way sensitivity analyses, where structural and parametric sensitivity analyses comprised 557 and 679 scenarios, respectively (Table 3). The ranges of parametric uncertainties were arbitrarily selected in 48% of them (326 scenarios), followed by alternative sources (256 scenarios, 38%) and 95% CI (97 scenarios, 14%). The 95% CI was more likely to be employed in the relative effectiveness (36%), yet arbitrary values were frequently used in resource use (78%) and drug price (70%).

**TABLE 3:** The ranges of values used in the parametric sensitivity analysis of the 50 dossiers submitted to the Economic Sub-Committee for listing at the Korean National Health Insurance.

	Scenarios (%)			
	95% Confidence interval^1^	Alternative sources^2^	Arbitrary values^3^	Total
Utility	46	108	54	208 (31%)
Resource use	0	45	163	189 (28%)
Relative effectiveness	43	53	23	119 (18%)
Baseline risk	0	32	33	59 (9%)
Drug Price	0	12	28	65 (10%)
Other^4^	8	6	25	39 (6%)
Total	97 (14%)	256 (38%)	326 (48%)	679 (100%)

1 95% confidence intervals of the corresponding parameters were estimated from the clinical trials.

2 Values obtained from source(s) other than the base case were used for the sensitivity analysis.

3 Authors explore the ranges of the sensentivity analysis without clinical or statistical rationales, such as ± 10%.

4 Parameters relevant with specific treatment, such as the incidence of the adverse event, or hospitalization rate.

A single scenarios for the sensitivity analysis corresponds to a case where a single variable is varied by a single plausible range, and a paired case (i.e,., ± 20%) was defined as a single scenario.

Regarding structural uncertainties, the discounting rate (202 scenarios) was most frequently examined, followed by time horizon (130 scenarios), model assumptions (i.e., treatment duration, duration of the effectiveness, comparator, adjusting for cross-over design; 94 scenarios), extrapolation (95 scenarios), and patient characteristics (age, disease severity, weight, or race; 36 scenarios).

**FIGURE 1:**
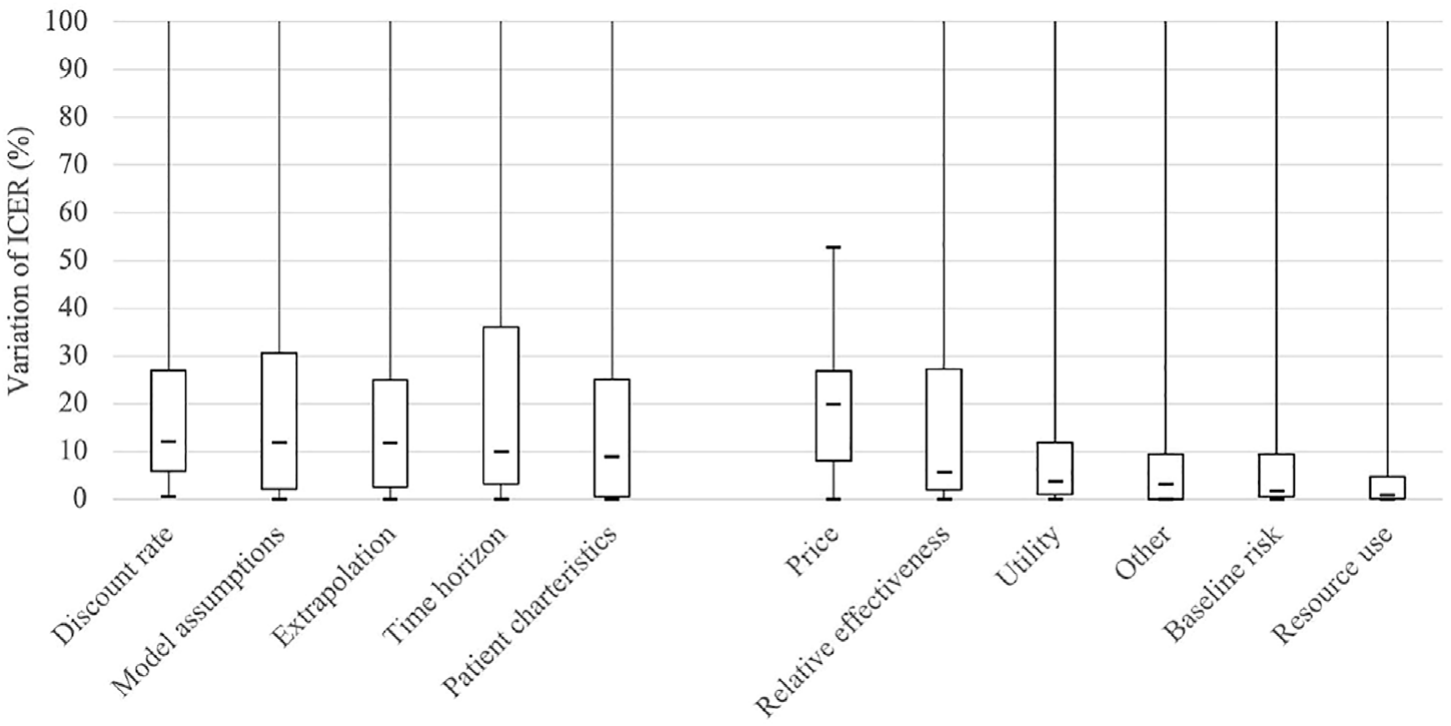
Boxplot comparing the variance of ICER (Incremental cost effectiveness ratio) for each scenario with reference to that of the base case. The “Other” implies parameters relevant with specific treatment, such as the incidence of the adverse event, or hospitalization rate.

The relative variance of ICER for each variable is presented in Figure 1 as box plots, where the distributions of each variable are skewed. In general, structural uncertainties showed wider interquartile ranges, compared with parametric uncertainties. The median of the relative variances in terms of percentage indicates that drug price has the highest impact (19.9%), followed by discount rate (12.2%), model assumptions (11.9%), extrapolation (11.8%), and time horizon (10.0%), suggesting that the most frequently examined variables do not always have the highest level of uncertainty. As shown in Figure 1, the median value of the percentage change is within 10% for most variables, excluding drug price, discount rate, model assumption, and extrapolation.

## Discussion

This study examined how uncertainty was explored in economic evaluations submitted for coverage decision-making in South Korea. The second version of the PE guidelines required that DSA be performed on all uncertain variables and encouraged submitters to conduct PSA for parametric uncertainty (Bae et al., 2013). When analyzing the submissions, 49 out of 50 cases, including a case of CMA, presented the results of DSA, and 18 of them additionally presented PSA.

Although most cases involved DSA, the assessment of uncertainty was somewhat limited. Relative effectiveness is one of the most critical parameters in cost-effectiveness analysis, yet only 52% of submissions conducted sensitivity analysis. Even though most of the submitted cases (48 out of 50) were analyzed using the model, only 58% of them performed sensitivity analysis on the model assumptions, and 4 cases did not perform DSA for utility among 46 cases that performed a cost-utility analysis (data not shown). In addition, 8 cases did not submit the DSA for the extrapolated model, even though survival analysis was performed.

The selection of the structural aspect of the model is an important decision that determines the model’s predictability (Afzali and Karnon, 2015). According to previous studies, the impact of structural uncertainty is similar to that of parametric uncertainty (Kim and Thompson, 2010; Frederix et al., 2014). However, it is known to be insufficiently reviewed compared to parametric uncertainty (Afzali and Karnon, 2015). Ghabri et al. (2016) reported that, as a result of reviewing manufacturers’ submissions to the French National Authority for Health (HAS), structural uncertainty was less frequently explored than methodological or parametric uncertainty, consistent with our assumptions (Ghabri et al., 2016). According to the analysis results of this study, however, there is no basis for concluding that structural uncertainty is more overlooked than parametric uncertainty, even though the term “structural uncertainty” is more widely defined in this study as including both methodological and structural uncertainty.

As shown in Figure 1, which shows the impact of each variable on the ICER, the median value of the percentage change is within 10%, excluding drug price, discount rate, model assumption, and extrapolation, which is smaller than the variances estimated in previous studies (Frederix et al., 2014; Kearns et al., 2020). Frederix et al. (2014) found that the ICER varied by 2–3 times depending on the difference in the structural aspects of the model and its parameterization (Frederix et al., 2014). In Kearns et al. (2000), it was confirmed that the ICER changed by 46.2% when different extrapolation methods were used (Kearns et al., 2020). Among the applications reviewed in this study, however, the median percentage change of ICER was 11.8%, and the upper quartile was only 24.9% in the cases where the sensitivity analysis was performed for the extrapolation method. Even considering that Kearns' study used a hypothetical dataset, it is questionable whether pharmaceutical companies have performed sensitivity analysis over a sufficient range.

A clear criterion such as 95% CI is used for only 14% of the sensitivity analyses, most of which are for relative efficacy. Arbitrary values or values cited from other studies are used in most cases. Even when published sources were cited, it is not easy to assess whether DSA was performed within a plausible range unless these sources were searched systematically. According to Ghabri et al. (2016), 43% of the submissions to HAS also lacked justification for the plausible range surrounding the point estimate of the parameter (Ghabri et al., 2016), which is similar to what we have observed in our analysis (48%).

Generally, high uncertainty has a negative impact on the reimbursement recommendation. Although our data do not provide any information about the association between the uncertainty and reimbursement decision, the authors’ experience of participating in the economic subcommittee of PBCAC suggested that when the uncertainty has a significant impact on the results, additional data is requested or negative appraisals are made. In this case, pharmaceutical companies are likely to be tempted to report with reduced uncertainty.

Therefore, when performing or reviewing sensitivity analysis, it is necessary to check the plausibility of the range used for sensitivity analysis. It is most desirable to determine the range through a systematic approach such as 95% CI. When such information is not available, systematically reviewing the existing literature is generally recommended to obtain a plausible range (Australian Government: Department of Health, 2016; The Canadian Agency for Drugs and Technologies in Health, 2017; The National Institute for Health and Care Excellence (NICE), 2013). If there is no proper prior research, it is necessary to seek expert opinions in a systematic way and set the range based on this.

From Figure 1, it is apparent that the influence of the variables related to the long-term effect is relatively large, except for the drug price, which pharmaceutical companies can strategically select. The discount rate, time horizon, and extrapolation are all in this case. In estimating the long-term effect based on short-term observations, the results vary greatly depending on the model assumptions, particularly the assumptions about the effect after the observation period. Accordingly, each country’s guidelines focus on the uncertainty that long-term extrapolation may have. Korea also emphasizes this point, as it revised the guidelines in 2021.

Similarly, 57% of submissions to the French HAS had the problem of unfounded extrapolation beyond the clinical trial (Ghabri et al., 2016). Masucci et al. (2017) also reported that the time horizon (56%) and model structure (36%) were frequently discussed by the economic reviewers of the pan-Canadian Oncology Drug Review (Masucci et al., 2017).

Recently, as drugs claiming long-term effects such as immune therapy and advanced therapy medicinal products have appeared, it is becoming more critical to evaluate the uncertainty in estimating long-term effects (Jönsson et al., 2019; Huygens et al., 2021) Due to insufficient patients or ethical reasons, new drugs used for rare severe diseases are often authorized based on a single-arm study rather than a randomized controlled trial. Additionally, the evidence for long-term effects is often uncertain because survival data are immature, along with other reasons. However, due to social pressure for early access, approvals or reimbursement decisions for these drugs are often made with very high uncertainty about clinical benefits (Grimm et al., 2020; Huygens et al., 2021). According to Kim and Prasad (2015), who followed up on the survival improvement of drugs approved based on the surrogate endpoint at the time of FDA approval (median follow-up 4.4 years), only 5 out of 36 cases demonstrated survival gain (Kim and Prasad, 2015). However, few efforts have been made to assess the validity of survival predictions compared to actual data (Latimner, 2013; Vickers, 2019).

In previous studies, several methods for exploring and managing uncertainty regarding long-term effects have been proposed, such as developing more specific guidance on exploring uncertainty surrounding extrapolation, requiring to follow up data after entry, using mature external data, or combining observed survival data with expert opinion in estimating long-term survival (Frederix et al., 2014; Cope et al., 2019; Huygens et al., 2021).

This study has several limitations. By reviewing the sensitivity analysis included in the first report submitted by the pharmaceutical company, we analyzed which variables were subjected to sensitivity analysis and their impact on ICER. However, no qualitative evaluation was performed to determine whether the range of values subjected to sensitivity analysis for each variable was appropriate. Moreover, the values identified in this study are those included in the first report and may differ from those used in the committee’s final deliberation. PSA was not reviewed in detail in this study because it was not a mandatory requirement for the study period, and the intention of this study is to confirm which variables were reviewed for structural and parametric uncertainties and the impact of each uncertain variable.

This study is the first attempt to explore the uncertainty in economic evaluations submitted for reimbursement decision-making in South Korea. Several studies explored the impact of uncertainty in economic evaluations, but only a few examined actual documents submitted to the HTA agencies. Given the growing importance of uncertainty, by reviewing how pharmaceutical companies are handling uncertainty in submissions to relevant authorities, we can find implications for what points should be emphasized to better address the uncertainty in cost-effectiveness. In particular, the fact that the ICER variation in this study was smaller than what was reported in the previous studies suggests that more prescribed guidance is necessary. Further study is necessary to assess the real impact of uncertainty in terms of the difference between what was predicted at the time of listing and how they actually performed in the follow-up studies.

## Conclusion

Most dossiers submitted to the committee for reimbursement decisions presented DSA results as suggested in the guidelines. However, considering the variance of ICER, in terms of the impact of each uncertainty, variability was not significant in most scenarios, which raises doubts as to whether the uncertainty evaluation was carried out within a sufficiently plausible range for each variable. Specific guidance regarding the ranges of the sensitivity analysis is necessary. Long-term benefits are often modeled based on uncertain short-term clinical data; therefore, the evaluation and management of uncertainties become more critical than before.

## Data Availability

The original contributions presented in the study are included in the article/Supplementary Material, further inquiries can be directed to the corresponding author.
