# Supplementary material for: How Sensitive is Sensitivity Analysis?: Evaluation of Pharmacoeconomic Submissions in Korea
Source: Front Pharmacol. 2022 May 16;13:884769. doi: 10.3389/fphar.2022.884769 (PMC9149282; doi:10.3389/fphar.2022.884769)
Supplement: Supplementary file 1 [file Table1.docx]

Supplementary Material

**Table A.** The variance of ICER (Incremental cost effectiveness ratio) for each scenario with reference to that of the base case.

|  | Number of scenarios | Relative variance of ICERs (distribution) | | | |
| --- | --- | --- | --- | --- | --- |
|  |  | Q1 | Median | Q3 | Mean |
| **Structural uncertainties** | |  |  |  |  |
| Discount rate | 202 | 5.9 | 12.2 | 27.0 | 19.6 |
| Model assumptions | 94 | 2.1 | 11.9 | 30.6 | 31.9 |
| Extrapolation | 95 | 2.5 | 11.8 | 24.9 | 24.8 |
| Time horizon | 130 | 3.3 | 10.0 | 36.1 | 46.4 |
| Patient characteristics | 36 | 0.5 | 9.0 | 25.1 | 24.4 |
| **Parametric uncertainties** | |  |  |  |  |
| Price | 40 | 8.1 | 19.9 | 26.9 | 19.8 |
| Relative effectiveness | 119 | 2.0 | 5.8 | 27.3 | 57.0 |
| Utility | 208 | 1.0 | 3.9 | 11.9 | 14.9 |
| Other^1^ | 39 | 0.0 | 3.2 | 9.4 | 35.5 |
| Baseline risk | 65 | 0.6 | 1.8 | 9.4 | 19.5 |
| Resource use | 208 | 0.1 | 1.0 | 4.7 | 10.0 |

^1^Parameters relevant with specific treatment, such as the incidence of the adverse event, or hospitalization rate.

A single scenarios for the sensitivity analysis corresponds to a case when a single variable is varied by a single plausible range, and a paired case (i.e, ±20%) was defined as a single scenario.

The variance of the ICER related with the sensitivity analyses were measured in percent (|ICER _sensitivity analysis_ - ICER _base case_|*100/ ICER _base case_) where paired (±95% CI) values were estimated as follows: (ICER _Max_ - ICER _min_)*100/ (2*ICER _base case_ )
